# Immune Checkpoint Inhibitor-Related Pancreatic Injury in Patients with Lung Cancer: Diagnostic Challenges, Multidisciplinary Management, and Outcomes

**DOI:** 10.3390/cancers18182930

**Published:** 2026-09-10

**Authors:** Aleksandra Piórek, Małgorzata Symonides, Marta Bijak-Ulejczyk, Dariusz Mirosław Kowalski, Maciej Krzakowski

**Affiliations:** 1 Department of Lung Cancer and Thoracic Tumors, Maria Skłodowska-Curie National Research Institute of Oncology, W.K. Roentgena 5, 02-781 Warsaw, Poland; dariusz.kowalski@nio.gov.pl (D.M.K.); maciej.krzakowski@nio.gov.pl (M.K.); 2 Clinical Department of Anesthesiology and Intensive Care, Maria Skłodowska-Curie National Research Institute of Oncology, W.K. Roentgena 5, 02-781 Warsaw, Poland

**Keywords:** immune checkpoint inhibitors, pancreatic injury, acute pancreatitis, immune-related adverse events, lung cancer

## Abstract

**Background/Objectives:** Immune checkpoint inhibitors (ICIs) can cause pancreatic immune-related adverse events ranging from isolated pancreatic enzyme elevation to severe acute pancreatitis. This study aimed to describe the clinical presentation, diagnostic classification, management, and outcomes of ICI-related pancreatic injury in patients with lung cancer. **Methods:** We conducted a retrospective, single-center case series of six patients with lung cancer who developed pancreatic injury during or after ICI treatment between January 2016 and December 2024. Clinical characteristics, laboratory and imaging findings, toxicity grade, management, and outcomes were extracted from the available medical records and analyzed descriptively. **Results:** The median time from ICI initiation to pancreatic injury was 8.6 months (range, 2.3–16.4 months). The recorded Common Terminology Criteria for Adverse Events (CTCAE) severity grade was 2 in two patients, 3 in one patient, and 4 in three patients; pancreatic enzyme elevations were evaluated separately from clinical pancreatic adverse-event severity. Five patients required hospitalization and received supportive care, including intravenous fluids and corticosteroids. One patient received mycophenolate mofetil, and one developed acute necrotizing pancreatitis requiring intensive care, endoscopic procedures, and repeated surgical interventions. Two patients died during follow-up. In one patient, death followed a prolonged course of severe necrotizing pancreatitis complicated by infected necrosis and multiorgan failure; in the second patient, the available retrospective records did not permit reliable attribution of the cause of death to pancreatic toxicity. Immunotherapy was resumed in two patients; recurrent pancreatic enzyme elevation occurred after rechallenge, but recurrent clinically overt pancreatitis was not documented. **Conclusions:** ICI-related pancreatic injury has a heterogeneous clinical presentation. Pancreatic enzyme elevation alone should not be considered equivalent to acute pancreatitis. Diagnostic classification requires integration of symptoms, biochemical findings, imaging, and exclusion of alternative etiologies. Management should be individualized, and severe cases may require early multidisciplinary involvement. The benefits of corticosteroids and routine pancreatic enzyme monitoring remain uncertain.

## Simple Summary

Immune checkpoint inhibitors are effective treatments for lung cancer but can rarely cause pancreatic injury. This injury may present only as an increase in pancreatic enzyme levels or may progress to severe acute pancreatitis. We retrospectively described six patients with lung cancer who developed pancreatic injury during or after treatment with immune checkpoint inhibitors. The clinical presentation and severity varied considerably. Five patients required hospitalization, and one developed necrotizing pancreatitis requiring intensive care, endoscopic procedures, and repeated surgery. Two patients resumed or continued immunotherapy after improvement; one subsequently developed recurrent asymptomatic pancreatic enzyme elevation without recurrent clinically overt pancreatitis. These observations emphasize that increased pancreatic enzyme levels should not automatically be considered equivalent to acute pancreatitis. Diagnosis should integrate symptoms, laboratory findings, imaging, and assessment of alternative causes. Severe cases may require early cooperation between oncologists, gastroenterologists, intensivists, radiologists, endoscopists, and surgeons.

## 1. Introduction

Immune checkpoint inhibitors (ICIs), including antibodies targeting cytotoxic T-lymphocyte-associated protein 4 (CTLA-4), programmed cell death protein 1 (PD-1), and programmed death ligand 1 (PD-L1), have become an essential component of treatment for several malignancies, including non-small-cell and small-cell lung cancer. By restoring antitumor T-cell activity, ICIs can provide durable clinical benefit; however, they may also disrupt immune tolerance and cause immune-related adverse events (irAEs) involving almost any organ system. Gastrointestinal and hepatobiliary toxicities are well recognized, whereas pancreatic adverse events remain uncommon and less well characterized [1,2].

Immune-related pancreatic toxicity encompasses a heterogeneous spectrum ranging from an isolated, asymptomatic elevation of serum amylase or lipase to clinically apparent acute pancreatitis and, rarely, severe necrotizing pancreatitis. Its reported incidence varies considerably because previous studies have used different definitions and have not consistently distinguished biochemical abnormalities from clinically confirmed acute pancreatitis. Pancreatic toxicity has been reported more frequently with combined anti-CTLA-4 and anti-PD-1/PD-L1 therapy than with ICI monotherapy [1,3]. Nevertheless, elevated pancreatic enzyme levels do not necessarily indicate acute pancreatitis. In a retrospective analysis of 119 patients receiving nivolumab plus ipilimumab, grade 3 or higher elevations of amylase and lipase were substantially more frequent than clinically confirmed pancreatitis, which occurred in only two patients [4]. Similarly, a meta-analysis of randomized trials demonstrated an increased risk of pancreatic enzyme elevation with combined ICI treatment but did not show a corresponding significant increase in clinically diagnosed pancreatitis compared with control therapy [3].

According to established diagnostic criteria, acute pancreatitis is diagnosed when at least two of the following are present: characteristic abdominal pain, serum amylase or lipase activity exceeding three times the upper limit of normal, and imaging findings consistent with pancreatic inflammation [5]. Consequently, the broader term “ICI-related pancreatic injury” may be more appropriate when these criteria are not fully met. The diagnosis remains one of exclusion and requires consideration of alternative etiologies, including alcohol use, gallstones or biliary sludge, hypertriglyceridemia, other medications, autoimmune pancreatitis, pancreatic metastases, and primary pancreatic neoplasms [1,2]. Evidence regarding optimal management is limited, particularly concerning the role of corticosteroids, the clinical significance of asymptomatic enzyme elevation, and the safety of resuming immunotherapy after recovery [1,2].

This study presents a retrospective single-center series of six patients with lung cancer who developed ICI-related pancreatic injury. We describe the clinical presentation, timing, severity, management, and outcomes of these events, including a case of fatal necrotizing pancreatitis requiring prolonged multidisciplinary treatment. By illustrating the wide spectrum of pancreatic toxicity associated with ICIs, this series aims to support appropriate diagnostic classification and individualized management of this rare but potentially life-threatening complication.

## 2. Materials and Methods

### 2.1. Study Design and Setting

This retrospective, single-center case series included patients with lung cancer treated with ICIs at the Maria Skłodowska-Curie National Research Institute of Oncology in Warsaw, Poland, between January 2016 and December 2024. Patients who developed pancreatic injury during or following ICI treatment were identified through a retrospective review of the available medical records. Because the total number of patients with lung cancer treated with ICIs during the study period was not available for this analysis, the incidence of ICI-related pancreatic injury was not estimated.

### 2.2. Case Definition and Eligibility Criteria

Patients were eligible for inclusion if they had a histologically confirmed diagnosis of lung cancer, had received at least one dose of an ICI, and subsequently developed pancreatic enzyme elevation and/or clinical or radiological findings considered compatible with pancreatic injury. For the purposes of this study, “ICI-related pancreatic injury” was used as an umbrella term encompassing both isolated pancreatic enzyme elevation and clinically apparent acute pancreatitis occurring in temporal association with ICI treatment.

Acute pancreatitis was distinguished from isolated biochemical pancreatic injury on the basis of the available clinical documentation. Established diagnostic criteria were considered, including characteristic abdominal pain, serum amylase or lipase activity exceeding three times the upper limit of normal, and imaging findings consistent with pancreatic inflammation; the presence of at least two of these features supported the diagnosis of acute pancreatitis [5]. The broader term “pancreatic injury” was retained when the available retrospective data did not permit uniform confirmation of these criteria.

Alternative causes of pancreatic injury were assessed using the information available in the medical records. Patients with a clearly established non-ICI etiology of pancreatic injury were excluded. Potential competing etiologies, including alcohol use, gallstone disease or biliary obstruction, hypertriglyceridemia, other medications, autoimmune pancreatitis, and malignancy-related pancreatic involvement, were considered when corresponding clinical, laboratory, or imaging data were available. Given the retrospective nature of the study, alternative etiologies could not be uniformly excluded in all patients. The relationship with ICI therapy was assessed on the basis of temporal association, clinical evaluation, and the exclusion of documented competing causes.

### 2.3. Data Collection

The following data were extracted from the medical records: age at the onset of pancreatic injury, sex, smoking history, Eastern Cooperative Oncology Group performance status, histological type and stage of lung cancer, and details of systemic anticancer treatment. ICI exposure was categorized as anti-CTLA-4 therapy, anti-PD-1/PD-L1 therapy, or combined anti-CTLA-4 and anti-PD-1/PD-L1 treatment. Concomitant administration of chemotherapy and the clinical setting of immunotherapy were also recorded.

Data relating to pancreatic injury included the time from ICI initiation to the onset of the event, presenting symptoms, serum amylase and lipase levels, available imaging findings, and the CTCAE severity grade recorded in the source medical documentation. Because the study period spanned the transition from CTCAE v4.03 to v5.0, the exact CTCAE version used contemporaneously was not consistently identifiable for every patient. However, the grading definitions for the CTCAE terms ‘pancreatitis’, ‘amylase increased’, and ‘lipase increased’ are unchanged between CTCAE v4.03 and v5.0. Pancreatic enzyme values were therefore reported separately from the recorded clinical severity grade and were not used interchangeably with the diagnosis or grade of pancreatitis. Where the original adverse-event term could be recovered, grading was interpreted according to that term; where it was not available, the grade was retained as recorded in the source database without retrospective reassignment based solely on enzyme values.

Management variables included hospitalization, intravenous fluid therapy, analgesia, corticosteroid treatment, additional immunosuppressive therapy, nutritional support, endoscopic procedures, surgical intervention, and intensive care unit admission. Recorded outcomes included complications of pancreatic injury, vital status at the last follow-up, temporary or permanent discontinuation of ICI therapy, resumption of immunotherapy after recovery, and subsequent recurrence of severe pancreatic toxicity. To facilitate patient-level interpretation of this case series, individual demographic, oncological, pancreatic toxicity, management, and outcome data were additionally summarized for each patient. For corticosteroid and additional immunosuppressive treatment, the specific agent, dose, route, duration, and tapering schedule were extracted when explicitly documented. These details were not retrospectively inferred when absent from the source records.

### 2.4. Statistical Analysis

Because of the small sample size and descriptive nature of the study, no formal hypothesis testing or comparative statistical analysis was performed. Continuous variables were summarized using the median and range, whereas categorical variables were reported descriptively. In addition to cohort-level summaries, patient-level clinical characteristics, treatment, pancreatic toxicity, management, and outcomes were presented in a dedicated table and clinical timeline. The patient with severe necrotizing pancreatitis was additionally presented as a detailed narrative case description.

### 2.5. Ethical Considerations

The study was conducted in accordance with the Declaration of Helsinki. The Institutional Review Board of the Maria Skłodowska-Curie National Research Institute of Oncology, Warsaw, Poland, reviewed the study and determined that formal ethical approval was not required because it involved a retrospective analysis of anonymized clinical data. The requirement for individual informed consent was waived in accordance with the Institutional Review Board’s determination. No directly identifying patient information or identifiable images were included in the analysis or manuscript.

## 3. Results

### 3.1. Patient Characteristics

**Figure 1:**
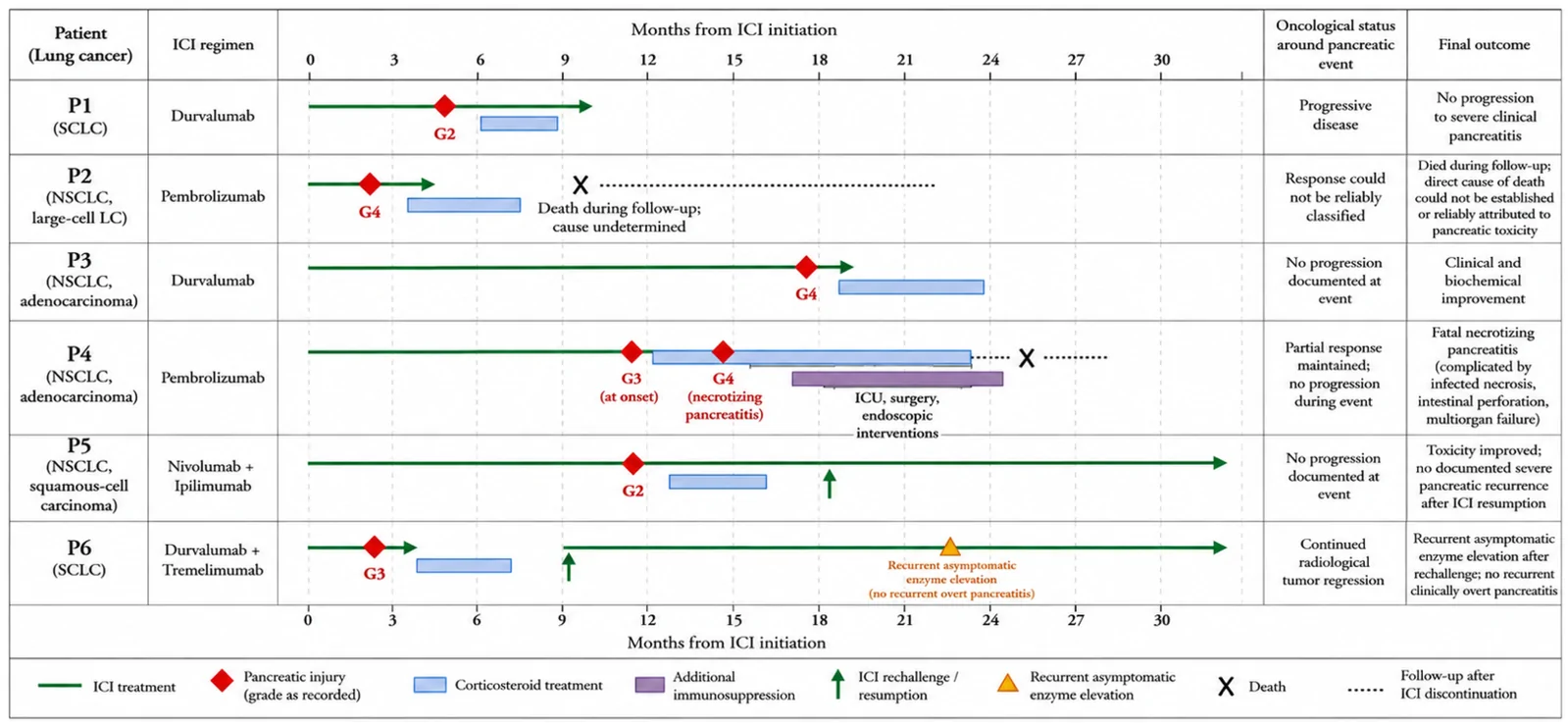
Patient-level timeline of immune checkpoint inhibitor treatment, pancreatic injury, management, and outcomes. Horizontal bars represent the clinical course from initiation of ICI therapy. Red diamonds indicate the onset of pancreatic injury. Recorded CTCAE severity grades are shown adjacent to each event. Corticosteroid treatment, additional immunosuppression, ICI resumption or continuation, recurrent pancreatic enzyme elevation, and final clinical outcomes are indicated where available. In Patient 4, acute pancreatitis was documented as grade 3 at onset and subsequently progressed to life-threatening grade 4 necrotizing pancreatitis. In Patient 6, recurrent asymptomatic pancreatic enzyme elevation occurred after ICI rechallenge without recurrent clinically overt pancreatitis. ICI, immune checkpoint inhibitor; CTCAE, Common Terminology Criteria for Adverse Events; ICU, intensive care unit; LC, lung carcinoma; NSCLC, non-small-cell lung cancer; SCLC, small-cell lung cancer.

Six patients with lung cancer who developed ICI-related pancreatic injury between January 2016 and December 2024 were included. The median age at the onset of pancreatic injury was 64 years (range, 44–75 years), four patients were male, and all were current or former smokers. Four patients had non-small-cell lung cancer and two had small-cell lung cancer. Two patients had stage III disease and four had stage IV disease. Four patients received an ICI in combination with chemotherapy, one received ICI monotherapy in the palliative setting, and one received consolidation immunotherapy. Four patients received anti-PD-1 or anti-PD-L1 treatment without CTLA-4 blockade, whereas two received combined anti-CTLA-4 and anti-PD-1/PD-L1 therapy. None of the patients received anti-CTLA-4 monotherapy. Baseline demographic, clinical, and treatment characteristics of the cohort are summarized in Table 1. Individual patient characteristics, ICI exposure, timing and presentation of pancreatic injury, management, and clinical outcomes are presented in Table 2 and Figure 1.

### 3.2. Onset and Severity of Pancreatic Injury

The median time from ICI initiation to the onset of pancreatic injury was 8.6 months (range, 2.3–16.4 months). Pancreatic enzyme elevation was documented at the onset of the event. In one patient, the maximum recorded amylase and lipase levels were 3807 U/L and 2203 U/L, respectively.

The recorded CTCAE severity grade was grade 2 in two patients, grade 3 in one patient, and grade 4 in three patients. These grades represent the clinical pancreatic adverse-event severity recorded in the source data and should not be interpreted as grades of isolated amylase or lipase elevation. The exact contemporaneous CTCAE adverse-event term was not recoverable for every patient. In the patient with severe necrotizing pancreatitis, acute pancreatitis was explicitly documented as grade 3 at onset and subsequently progressed to life-threatening grade 4 disease. Because the available retrospective documentation did not permit uniform confirmation of the diagnostic criteria for acute pancreatitis in every patient, these events are collectively reported as ICI-related pancreatic injury. One patient experienced an additional grade 3 or higher immune-related adverse event involving the skin.

### 3.3. Management and Outcomes

Five patients received corticosteroid treatment, although detailed dosing and tapering schedules could not be reconstructed uniformly from the retrospective records. In the patient with severe necrotizing pancreatitis, methylprednisolone was administered intravenously at 100 mg once daily and was subsequently tapered to 40 mg once daily. Because of an insufficient clinical response, mycophenolate mofetil was subsequently introduced; however, the exact administered dose and duration could not be reliably reconstructed. In another patient, recurrent asymptomatic pancreatic enzyme elevation after ICI resumption was treated with oral methylprednisolone 64 mg/day for one week. For the remaining corticosteroid-treated patients, the medical records identified the corticosteroid used but did not consistently document the initial dose, route, treatment duration, or tapering schedule. Corticosteroid treatment was individualized by the treating physicians rather than administered according to a prespecified institutional protocol. Where detailed dosing information was available, the regimens were broadly consistent with contemporary guideline-based management of clinically significant irAEs. One patient developed acute necrotizing pancreatitis requiring intensive care, endoscopic procedures, and repeated surgical interventions.

At the last follow-up, two patients had died and four were alive. One death occurred after a prolonged clinical course of severe necrotizing pancreatitis complicated by infected pancreatic and peripancreatic necrosis, intestinal perforation, repeated surgical interventions, and multiorgan failure, as described in detail below. The second patient had developed symptomatic pancreatic toxicity during pembrolizumab treatment, with peak amylase and lipase activities of 579 U/L and 1303 U/L, respectively, and was treated with corticosteroids. She subsequently died during follow-up; however, the available retrospective documentation was insufficient to establish the direct cause of death or to reliably attribute it to pancreatic toxicity. No post-mortem examination was available. Two patients resumed ICI therapy after improvement of pancreatic toxicity. In Patient 5, nivolumab plus ipilimumab was resumed on 14 August 2018 after clinical improvement and corticosteroid treatment, and nivolumab was subsequently continued. In December 2018, recurrent grade 3 elevations of amylase and lipase were documented, accompanied by nausea and reduced appetite but without abdominal pain or documented recurrent clinically overt acute pancreatitis. Corticosteroid treatment was reintroduced. In Patient 6, ICI therapy was also resumed and was subsequently followed by recurrent asymptomatic pancreatic enzyme elevation without documented recurrent clinically overt pancreatitis. No new non-pancreatic irAEs were documented after rechallenge in either patient. In Patient 5, a transient grade 2 febrile episode considered probably related to nivolumab was recorded after treatment resumption, but it was not formally classified as an immune-related adverse event in the source documentation. Oncological status around the time of pancreatic injury varied across the cohort. Progressive disease was documented in one patient. One patient with severe necrotizing pancreatitis remained in partial response without evidence of oncological progression, and continued radiological tumor regression was documented in another patient. In two additional patients, no disease progression was documented at the time of pancreatic injury, whereas the oncological response of one patient could not be reliably classified from the available retrospective records. Individual oncological outcomes are summarized in Figure 1.

### 3.4. Detailed Clinical Course of Severe Necrotizing Pancreatitis

A man in his late 60s was diagnosed with stage IV adenocarcinoma of the left lung with bilateral pulmonary metastases in August 2023. Next-generation sequencing identified no actionable molecular alterations, and the tumor proportion score for PD-L1 expression was 15%. The patient was a heavy smoker and had a history of hypertension and chronic obstructive pulmonary disease. His Eastern Cooperative Oncology Group performance status was 0 before the onset of pancreatic toxicity. The clinical course of this patient is included in the patient-level timeline presented in Figure 1.

In September 2023, he started combination chemoimmunotherapy comprising pembrolizumab and platinum-based chemotherapy. Treatment resulted in a partial response, which was documented in July 2024. In September 2024, approximately one year after ICI initiation, the patient developed symptoms consistent with acute pancreatitis. Serum amylase and lipase activities exceeded three times the upper limit of normal, and imaging demonstrated pancreatic inflammation and edema without evidence of necrosis. The presence of compatible symptoms, pancreatic enzyme elevation, and characteristic imaging findings supported the diagnosis of acute pancreatitis. Representative serial CT images illustrating the radiological evolution of pancreatic and peripancreatic changes are provided in Supplementary Figure S1.

The patient was hospitalized and initially treated with intravenous fluids, opioid analgesia, nutritional support, and intravenous methylprednisolone, initially administered at 100 mg once daily and subsequently tapered to 40 mg once daily. Because of an insufficient clinical response, mycophenolate mofetil was subsequently introduced. The exact administered dose and duration of mycophenolate mofetil could not be reconstructed reliably from the available retrospective records. Despite an initial reduction in pancreatic enzyme levels, his clinical condition deteriorated. In October 2024, he underwent emergency surgery for abdominal abscesses associated with acute necrotizing pancreatitis and was admitted to the intensive care unit.

The subsequent clinical course was complicated by intestinal perforation, infected pancreatic and peripancreatic necrosis, and persistent intra-abdominal and retroperitoneal fluid collections. The patient required repeated laparotomies, surgical debridement, drainage, peritoneal lavage, and vacuum-assisted abdominal closure. Cultures of peritoneal fluid and blood yielded *Streptococcus anginosus* and *Pseudomonas aeruginosa*, and antimicrobial treatment was adjusted according to susceptibility results. Mechanical ventilation, parenteral nutrition, and prolonged intensive care support were required.

During the intensive care unit stay, the patient developed progressive renal and coagulation abnormalities, gastrointestinal bleeding, and metabolic encephalopathy. Renal replacement therapy was initiated because of kidney failure. Persistent pancreatic fluid leakage through the abdominal drains prompted endoscopic retrograde cholangiopancreatography. The first attempt to place a pancreatic duct stent was unsuccessful; however, stent placement was achieved during a subsequent procedure.

Despite maximal supportive treatment and repeated surgical and endoscopic interventions, the patient developed progressive multiorgan dysfunction. In late November 2024, he required high-dose vasopressor support. Computed tomography angiography excluded pulmonary embolism, while abdominal imaging demonstrated perfusion abnormalities without substantial improvement in the pancreatic and abdominal complications. The lung cancer remained in partial response, with no evidence of oncological progression. The patient died after approximately two months of hospitalization.

## 4. Discussion

This retrospective case series illustrates the broad clinical spectrum of ICI-related pancreatic injury in patients with lung cancer, ranging from pancreatic enzyme elevation to life-threatening necrotizing pancreatitis. The principal findings were the marked heterogeneity of presentation and severity, the relatively delayed onset of pancreatic toxicity in some patients, the need for hospitalization in five of six cases, and the occurrence of severe complications in two patients. Two patients were able to resume immunotherapy after resolution of the pancreatic event without further severe pancreatic toxicity. However, because this study did not include the total population of patients treated at our institution, it cannot provide an estimate of incidence or identify factors associated with the development or severity of pancreatic injury.

Several observational studies and meta-analyses have reported an association between the development of irAEs and improved outcomes with ICI therapy, including higher objective response rates and longer progression-free and overall survival in patients with lung cancer. However, these observations apply to irAEs as a heterogeneous group and should not be extrapolated directly to pancreatic toxicity. In the present case series, oncological outcomes were heterogeneous: one patient had progressive disease at the time of pancreatic injury, whereas other patients had ongoing tumor control, including documented partial response or continued radiological regression. Given the very small sample size, retrospective design, and absence of a comparator group, our study cannot evaluate whether pancreatic irAEs are associated with enhanced antitumor efficacy [6].

### 4.1. Incidence and Spectrum of Pancreatic Toxicity

Reported estimates of ICI-related pancreatic toxicity vary substantially because studies have used different definitions and have frequently combined asymptomatic pancreatic enzyme elevation with clinically confirmed acute pancreatitis. In a systematic review and meta-analysis of 50 randomized trials involving 35,223 patients, the pooled incidence of pancreatic injury, defined as pancreatitis or amylase/lipase elevation, was 2.22%. The incidence was higher with combined ICI treatment than with single-agent therapy (3.76% versus 2.25%) [7]. These estimates should not be interpreted as the incidence of clinical acute pancreatitis because laboratory abnormalities constituted a substantial proportion of the reported events.

The distinction between biochemical pancreatic injury and acute pancreatitis is particularly relevant in lung cancer. In a 2024 case series of 21 patients with lung cancer, 22 pancreas-specific irAEs were identified: 13 were classified as pancreatic injury, four as acute pancreatitis, and five as ICI-related diabetes mellitus [8]. This classification supports the use of “ICI-related pancreatic injury” as an umbrella term and demonstrates that pancreatic irAEs may involve exocrine inflammation, isolated enzyme abnormalities, or endocrine dysfunction. In our series, the available retrospective documentation did not allow uniform confirmation of conventional acute pancreatitis criteria in every patient; therefore, classifying all six events as pancreatitis would risk overdiagnosis.

The median time from ICI initiation to pancreatic injury in our cohort was 8.6 months, with a range of 2.3–16.4 months. This interval was longer than the median of 12 weeks reported in the lung cancer series by Liu et al. [8] and the median of 108 days reported in a 2025 systematic review of published ICI-associated pancreatitis cases [9]. However, both previous studies demonstrated wide variability in the time to onset. Collectively, these observations indicate that pancreatic toxicity may develop not only early during treatment but also after prolonged ICI exposure. Differences between studies may also reflect variation in enzyme-monitoring practices and in the definitions used to identify the onset of pancreatic injury.

### 4.2. Clinical Presentation and Diagnostic Classification

Pancreatic enzyme elevation during ICI treatment does not necessarily indicate acute pancreatitis. This distinction was also demonstrated by Michot et al., who reported that only 3 of 21 patients with ICI-associated lipase elevation had clinical or radiological evidence of pancreatitis, whereas most enzyme elevations were not associated with clinically significant pancreatic disease [10]. In a retrospective analysis of 119 patients receiving nivolumab plus ipilimumab, grade 3 or higher elevations of amylase and lipase occurred in 8.4% and 26.9% of patients, respectively, whereas clinically confirmed pancreatitis occurred in only two patients (1.7%) [4]. Similar discrepancies between biochemical abnormalities and clinical pancreatitis have been reported in subsequent reviews [7].

According to established diagnostic criteria, acute pancreatitis should be diagnosed when at least two of three features are present: characteristic abdominal pain, serum amylase or lipase activity more than three times the upper limit of normal, and characteristic imaging findings [4,5]. CTCAE grading should be interpreted separately from conventional diagnostic criteria for acute pancreatitis. CTCAE includes distinct adverse-event terms for pancreatitis, amylase increased, and lipase increased, and a high-grade laboratory abnormality does not necessarily indicate high-grade clinical pancreatitis [7]. This distinction is particularly relevant in our cohort because pancreatic enzyme values and clinical severity were not concordant in all patients. Consequently, enzyme activities are reported separately from the clinical CTCAE severity grades recorded in the source documentation. In particular, a CTCAE category based on enzyme elevation or radiological findings alone does not necessarily confirm clinical acute pancreatitis. This distinction is important in our cohort because the available medical records supported different levels of diagnostic certainty among individual patients.

ICI-related pancreatic injury remains a diagnosis of exclusion. Relevant alternative causes include gallstones or biliary sludge, alcohol use, hypertriglyceridemia, hypercalcemia, other medications, pancreatic metastases, pancreatic or periampullary neoplasms, and autoimmune pancreatitis [2]. The diagnostic evaluation should be guided by symptoms and should include appropriate biochemical testing and imaging. Histological confirmation is rarely available. ICI-associated pancreatic injury has also been proposed as a distinct form of drug-induced immune-mediated pancreatic disease, termed type 3 autoimmune pancreatitis. This entity may encompass asymptomatic enzyme elevation as well as clinically apparent pancreatitis, and pancreatic imaging may be normal in some patients [11]. Consequently, the causal relationship between ICI exposure and pancreatic injury in this cohort was based on temporal association, clinical assessment, and the exclusion of documented alternative etiologies.

Most patients with biochemical pancreatic injury have normal pancreatic imaging. When abnormalities are present, the reported findings include pancreatic enlargement, heterogeneous or reduced enhancement, peripancreatic fat stranding, and fluid collections [8]. The patient with the most severe course in our series initially had pancreatic inflammation and edema without necrosis but subsequently developed necrotizing pancreatitis, infected collections, intestinal perforation, and multiorgan dysfunction. This case illustrates that an initially non-necrotizing radiological presentation may precede the development of severe complications.

### 4.3. Management and the Role of Corticosteroids

Management should first follow the principles applied to acute pancreatitis of other etiologies, including appropriate intravenous fluid therapy, analgesia, correction of electrolyte and metabolic abnormalities, and nutritional support [5]. Current evidence favors controlled, clinically guided fluid resuscitation rather than indiscriminate aggressive hydration, because excessive fluid administration may increase the risk of fluid overload without improving outcomes. Enteral nutrition should be introduced when feasible, while antibiotics should be reserved for documented or strongly suspected infection. Endoscopic or surgical interventions should be guided by established indications such as ductal disruption, biliary obstruction, infected necrosis, perforation, or persistent symptomatic collections [5].

The role of corticosteroids in ICI-related pancreatic injury remains uncertain. Although corticosteroid use in this series was individualized, the available dosing data were broadly consistent with general ASCO recommendations for clinically significant irAEs. Importantly, however, ASCO notes that the role of corticosteroids specifically in ICI-associated pancreatitis remains insufficiently defined [12]. In the retrospective study by Abu-Sbeih et al., corticosteroid treatment was not associated with a shorter duration of symptoms, faster improvement in pancreatic enzyme levels, fewer long-term complications, or improved overall survival [1]. In contrast, corticosteroids have been used frequently in published cases of clinically apparent ICI-related pancreatitis. In the 2025 systematic review, 73.8% of patients received corticosteroids, but the case-based nature of the evidence and the absence of an appropriate control group prevent conclusions regarding treatment efficacy [9]. Similarly, the lung cancer case series reported clinical recovery after supportive treatment with or without corticosteroids, but it could not establish a causal benefit from immunosuppression [8].

Therefore, corticosteroid therapy should not be considered automatically indicated for isolated asymptomatic enzyme elevation. In patients with symptomatic or severe pancreatic inflammation in whom an immune-mediated mechanism is considered likely, corticosteroids may be considered after exclusion of alternative causes, particularly when there are concurrent irAEs requiring immunosuppression [2,9]. The evidence supporting additional immunosuppressive agents in steroid-refractory pancreatic toxicity is limited to individual reports and extrapolation from other irAEs. In our severe case, mycophenolate mofetil was introduced because of an insufficient response to initial corticosteroid treatment; however, the subsequent progression to necrotizing pancreatitis does not allow its efficacy to be determined.

### 4.4. Monitoring and Resumption of Immunotherapy

Routine measurement of amylase and lipase in all asymptomatic patients receiving ICIs is not generally recommended because pancreatic enzyme elevations are relatively frequent, nonspecific, and poorly correlated with clinical pancreatitis [4]. Enzyme testing and pancreatic imaging are more appropriately performed in patients with suggestive symptoms, unexplained abdominal complaints, compatible radiological abnormalities, or clinical suspicion of pancreatic toxicity. Once pancreatic injury has been identified, follow-up should be individualized according to symptoms, biochemical abnormalities, imaging findings, severity, and response to treatment. Long-term pancreatic sequelae should also be considered. In a retrospective study, ICI-associated exocrine pancreatic insufficiency occurred as a delayed complication, and previous clinical pancreatitis was substantially more frequent among patients who subsequently developed exocrine insufficiency than among controls [13]. Monitoring for symptoms suggestive of exocrine pancreatic insufficiency and for disturbances of glucose metabolism may therefore be appropriate after clinically significant pancreatic injury.

The decision to withhold, discontinue, or resume immunotherapy should also be individualized. In the study by Abu-Sbeih et al., four of 35 patients who resumed ICI therapy developed recurrent lipase elevation, which was symptomatic in only one patient [1]. In the 2025 systematic review, seven patients were rechallenged without recurrence of pancreatitis, although the available evidence was derived from selected published cases [9]. In the lung cancer case series, most isolated pancreatic injuries resolved without specific treatment or permanent ICI discontinuation, whereas clinically apparent pancreatitis generally resulted in treatment interruption [8].

In our cohort, two patients resumed ICI therapy after improvement of pancreatic toxicity. In one patient, nivolumab plus ipilimumab was resumed after clinical recovery, and recurrent grade 3 pancreatic enzyme elevation was subsequently documented, accompanied by nausea and reduced appetite but without recurrent clinically overt acute pancreatitis. In the second patient, recurrent asymptomatic pancreatic enzyme elevation was documented after ICI resumption, also without recurrent clinically overt pancreatitis. These observations suggest that rechallenge may be feasible in selected patients after recovery from less severe pancreatic events, but they also demonstrate that recurrent biochemical pancreatic toxicity may occur. Given the very small number of rechallenged patients, our findings are insufficient to establish the general safety of this approach. Rechallenge should take into account the certainty of the initial diagnosis, severity and reversibility of pancreatic injury, presence of residual pancreatic dysfunction, availability of alternative anticancer treatment, previous oncological benefit, and patient preference. Permanent discontinuation is generally favored after life-threatening pancreatic toxicity, whereas resumption may be considered after complete clinical recovery from less severe events following multidisciplinary assessment. A recent review focused on ICI rechallenge in advanced-stage lung cancer similarly emphasized that rechallenge after irAEs should be individualized according to the type and severity of the initial toxicity, prior oncological benefit, available alternatives, and the risk of recurrence [14].

### 4.5. Severe and Fatal Pancreatic Toxicity

Although most pancreatic enzyme abnormalities are asymptomatic or reversible, clinically apparent ICI-related pancreatitis may occasionally be severe or fatal. A 2025 systematic review of 61 published patients reported grade 3–4 pancreatitis in 64.0%, improvement in 83.6%, recurrence in 8.2%, and death in 8.2% [9]. These figures are strongly influenced by publication bias because severe and unusual cases are more likely to be reported. Similarly, pharmacovigilance databases can identify safety signals but cannot provide true incidence rates because of selective reporting, missing denominators, duplicate reports, and incomplete clinical information.

Our detailed case illustrates the potential for progression from initially non-necrotizing pancreatitis to infected necrosis, intestinal perforation, pancreatic duct leakage, repeated surgical interventions, and multiorgan failure. The absence of radiological cancer progression and the persistence of partial oncological response support the conclusion that the terminal deterioration was predominantly related to abdominal and systemic complications rather than progression of lung cancer. Nevertheless, because of the retrospective nature of the analysis and the coexistence of infection, surgery, and critical illness, the contribution of ICI-mediated pancreatic inflammation to the final outcome cannot be quantified with certainty.

The severe clinical course also highlights the need for early multidisciplinary involvement. Collaboration between oncology, gastroenterology, intensive care, radiology, endoscopy, surgery, infectious diseases, and nutritional teams may be required when complications such as necrosis, infection, perforation, ductal disruption, bleeding, or organ failure occur.

### 4.6. Strengths and Limitations

The principal strength of this study is its focus on pancreatic toxicity in a population composed exclusively of patients with lung cancer and the presentation of events across a broad range of clinical severity. The detailed description of necrotizing pancreatitis provides additional information about a rare but potentially fatal course and its complex multidisciplinary management.

The study has several limitations. It was a retrospective, single-center case series including only six patients. Because the total number of patients with lung cancer treated with ICIs during the study period was not available, the incidence of ICI-related pancreatic injury could not be estimated. Complete clinical, biochemical, and imaging information required to retrospectively verify the conventional diagnostic criteria for acute pancreatitis was not available for all patients. No formal causality assessment algorithm was applied, and the relationship between ICI exposure and pancreatic injury was based on temporal association and the available exclusion of competing etiologies. Imaging findings were obtained from radiological reports available in the medical records, without a central reassessment of the original images. The small sample size and descriptive study design precluded formal statistical comparisons, identification of risk factors, and reliable comparisons between ICI regimens. Treatment strategies were heterogeneous and individually determined by the treating physicians; therefore, no conclusions can be drawn regarding the comparative effectiveness of corticosteroids, mycophenolate mofetil, supportive care, or ICI discontinuation. Long-term endocrine and exocrine pancreatic function was not systematically assessed in all patients; therefore, delayed pancreatic sequelae may have been underrecognized. The findings should consequently be interpreted as descriptive and hypothesis-generating.

Despite these limitations, the series emphasizes that ICI-related pancreatic toxicity should not be treated as a uniform diagnosis. Accurate distinction between isolated enzyme elevation, clinical acute pancreatitis, and other pancreatic manifestations is necessary to avoid both overdiagnosis and delayed recognition of potentially life-threatening disease.

## 5. Conclusions

ICI-related pancreatic injury is a heterogeneous toxicity that may range from isolated pancreatic enzyme elevation to clinically apparent acute pancreatitis and, rarely, life-threatening necrotizing disease. Pancreatic enzyme elevation alone should not be considered equivalent to acute pancreatitis; diagnosis requires integration of symptoms, biochemical findings, and imaging, together with exclusion of alternative etiologies. Management should follow established principles of acute pancreatitis care and should be individualized according to clinical severity. The benefit of corticosteroids remains uncertain, particularly in patients with asymptomatic biochemical abnormalities.

Routine pancreatic enzyme monitoring in asymptomatic patients receiving ICIs is not generally supported by the available evidence. After recovery from a less severe pancreatic event, resumption of immunotherapy may be considered in selected patients following an individualized multidisciplinary assessment of oncological benefit and the risk of recurrence. Life-threatening pancreatic toxicity generally warrants permanent ICI discontinuation. Although limited by its retrospective design and small sample size, this series highlights the importance of accurate diagnostic classification and early multidisciplinary management of severe pancreatic complications.

## Figures and Tables

**Table 1:** Demographic, clinical, and treatment characteristics of the study population (n = 6).

Characteristic	Value
Age at pancreatic injury, years, median (range)	64 (44–75)
Sex, n (%)	
Male	4 (66.7)
Female	2 (33.3)
Smoking history, n (%)	
Current smoker	4 (66.7)
Former smoker	2 (33.3)
ECOG performance status, n (%)	
0	2 (33.3)
1	4 (66.7)
Cancer type, n (%)	
NSCLC, adenocarcinoma	3 (50.0)
NSCLC, large-cell lung carcinoma	1 (16.7)
SCLC	2 (33.3)
Cancer stage, n (%)	
Stage III	2 (33.3)
Stage IV	4 (66.7)
ICI regimen, n (%)	
Anti-PD-1/PD-L1 without CTLA-4 blockade	4 (66.7)
Combined anti-CTLA-4 and anti-PD-1/PD-L1	2 (33.3)
ICI administered with chemotherapy, n (%)	4 (66.7)
Other grade ≥3 irAEs, n (%)	
Dermatologic	1 (16.7)

NSCLC, non-small-cell lung cancer; SCLC, small-cell lung cancer; ICI, immune checkpoint inhibitor; CTLA-4, cytotoxic T-lymphocyte-associated protein 4; PD-1, programmed cell death protein 1; PD-L1, programmed death ligand 1; irAE, immune-related adverse event.

**Table 2:** Individual clinical characteristics, pancreatic toxicity, management, and ICI resumption.

Patient	Lung Cancer	ICI Regimen	Time to Pancreatic Event, Months	Clinical Presentation	Peak Amylase, U/L	Peak Lipase, U/L	Recorded CTCAE Severity Grade	Management	ICI Resumption/Continuation
P1	SCLC	Durvalumab	5.6	Pancreatic injury; detailed symptom documentation incomplete	NR	NR	2	Detailed treatment regimen not reliably available	No documented rechallenge
P2	NSCLC, large-cell lung carcinoma	Pembrolizumab	2.3	Symptomatic pancreatic toxicity	579	1303	4	Methylprednisolone	No
P3	NSCLC, adenocarcinoma	Durvalumab	16.4	Symptomatic pancreatic toxicity	197	825	4	Methylprednisolone; detailed dose, route, duration, and taper not reliably available	No
P4	NSCLC, adenocarcinoma	Pembrolizumab	12.1	Acute pancreatitis progressing to severe necrotizing pancreatitis	3807	2203	4	IV methylprednisolone 100 mg/day, subsequently tapered to 40 mg/day; mycophenolate mofetil added; ICU, endoscopic and repeated surgical interventions	No
P5	NSCLC, adenocarcinoma	Nivolumab + ipilimumab	11.7	Symptomatic pancreatic toxicity; symptoms improved after corticosteroid treatment	261	801	2	Prednisone 80 mg/day orally, followed by gradual taper; at recurrent pancreatic enzyme elevation after rechallenge, intravenous methylprednisolone 125 mg was administered, followed by prednisone 100 mg/day orally	Yes; nivolumab + ipilimumab resumed on 14 August 2018; nivolumab subsequently continued
P6	SCLC	Durvalumab + tremelimumab	2.4	Symptomatic pancreatic toxicity	NR	NR	3	Methylprednisolone; subsequent recurrent asymptomatic enzyme elevation treated with oral methylprednisolone 64 mg/day for 1 week	Yes; ICI rechallenge/resumption followed by recurrent asymptomatic pancreatic enzyme elevation without recurrent clinically overt pancreatitis

CTCAE, Common Terminology Criteria for Adverse Events; ICI, immune checkpoint inhibitor; IV, intravenous; NR, not reliably available from the retrospective records; NSCLC, non-small-cell lung cancer; SCLC, small-cell lung cancer; ICU, intensive care unit.

## Data Availability

The data supporting the findings of this study are available from the corresponding author upon reasonable request. The data are not publicly available because they contain clinical information and are subject to institutional data-protection requirements.

## Supplementary Materials

The following supporting information can be downloaded at: https://www.mdpi.com/article/10.3390/cancers18182930/s1. Figure S1. Serial abdominal CT findings in the patient with severe necrotizing pancreatitis. (A) Contrast-enhanced abdominal CT obtained on 30 September 2024 showing pancreatic and peripancreatic inflammatory changes with associated fluid collections. (B) Contrast-enhanced abdominal CT obtained on 10 October 2024 at a comparable anatomical level, showing progression of pancreatic and peripancreatic inflammatory changes with complex gas-containing collections, consistent with complicated necrotizing pancreatitis.
