# Supplementary material for: Immune Checkpoint Inhibitor-Related Pancreatic Injury in Patients with Lung Cancer: Diagnostic Challenges, Multidisciplinary Management, and Outcomes
Source: Cancers (Basel). 2026 Sep 10;18(18):2930. doi: 10.3390/cancers18182930 (PMC13604156; doi:10.3390/cancers18182930)
Supplement: Supplementary file 1 [file cancers-18-02930-s001.zip › cancers-4508747-supplementary.pdf]

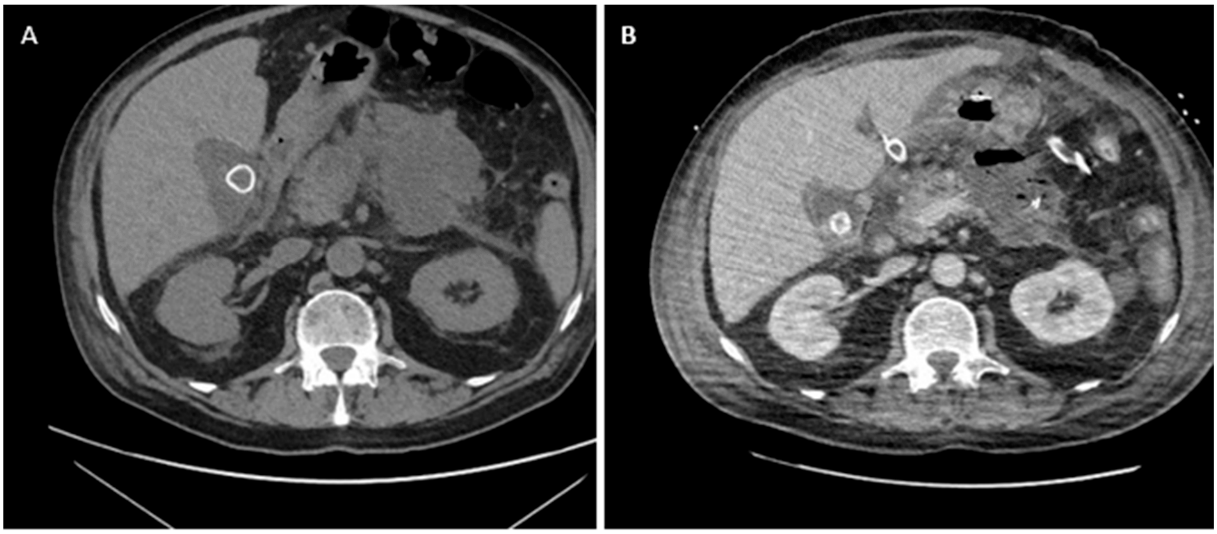

Supplementary Figure S1. Serial abdominal CT findings in the patient with severe necrotizing pancreatitis.

(A) Contrast-enhanced abdominal CT obtained on 30 September 2024 showing pancreatic and peripancreatic inflammatory changes with associated fluid collections.

(B) Contrast-enhanced abdominal CT obtained on 10 October 2024 at a comparable anatomical level, showing progression of pancreatic and peripancreatic inflammatory changes with complex gas-containing collections, consistent with complicated necrotizing pancreatitis.
